# Introduction

**DOI:** 10.1093/bib/bbaf631.fm001

**Published:** 2025-12-12

## INTRODUCTION

This Introduction summarizes the review and inclusion process for abstracts published in this special supplement of *Briefings in Bioinformatics*, which serves as the official Abstract Book for the 34th International Conference on Genome Informatics (GIW) and the 8th ISCB-Asia Conference (GIW ISCB-Asia 2025).

The conference was held in Hong Kong, China, from 10–13 December 2025, and brought together researchers from across Asia and around the world to share new developments and ideas in bioinformatics and computational biology.

## THE ABSTRACT REVIEW AND INCLUSION PROCESS

A total of 271 abstracts were submitted to GIW ISCB-Asia 2025, covering a broad spectrum of topics across bioinformatics and computational biology. Following review and author confirmations, 85 abstracts were collected for inclusions in this supplement. Talks are represented with extended two-page abstracts, while poster presentations appear as 300-word abstracts.

140 posters and 41 talks were accepted for presentation at the conference, resulting in an acceptance rate of 74% for posters and 15% for talks. The submissions were distributed across multiple tracks, including the GIW ISCB-Asia talk and poster sessions, as well as the 4th Asian Student Council Symposium. Each submission was evaluated for quality, clarity, and relevance, with 925 reviews completed by 45 external reviewers and a diverse international Programme Committee.

Accepted abstracts spanned a wide range of topics, including cutting-edge AI applications in biology and medicine, cancer genomics and personalized medicine, single-cell analysis, metagenomics and microbiome analysis, and multi-omic informatics, among others. Authors represented 38 countries or regions, with strong participation from China, Hong Kong, India, Japan, South Korea, the United States, and, reflecting the conference’s global reach and growing engagement throughout the Asia-Pacific region.

The high acceptance rate highlights the inclusive and collaborative nature of GIW ISCB-Asia, which aims to showcase the breadth of ongoing bioinformatics research while encouraging community-wide participation and knowledge sharing.

## ACKNOWLEDGEMENTS

We extend our sincere thanks to all authors who contributed their work to GIW ISCB-Asia 2025, and to both the School of Computing & Data Science and the School of Biomedical Sciences at The University of Hong Kong for hosting the conference this year. We are deeply grateful to the Area and Programme Committee members, external reviewers, and organizing committees for their time and effort in supporting the review and coordination process. We also thank ISCB and the editorial team at *Briefings in Bioinformatics* for their partnership in producing this supplement.

Together, these contributions have made GIW ISCB-Asia 2025 a dynamic and inspiring venue for collaboration and scientific exchange within the global bioinformatics community.

## STEERING COMMITTEE

- General Chair: Ruibang Luo, The University of Hong Kong
- Co-chair: Yuanhua Huang, The University of Hong Kong
- Co-chair: Limsoon Wong, National University of Singapore
- PC chair: Wing-Kin (Ken) Sung, The Chinese University of Hong Kong
- Secretary: Shumin Li, The University of Hong Kong

## SCIENTIFIC PROGRAMME COMMITTEE

- Chair: Wing-Kin (Ken) Sung, Chinese University of Hong Kong
- Co-Chair: Xiujie Wang, Chinese Academy of Sciences
- Co-Chair: Ruibang Luo, The University of Hong Kong

## AREA CHAIRS

- Lachlan Coin, University of Melbourne
- Huijuan Feng, Fudan University
- Chung-Chau Hon, RIKEN
- Jiandong Huang, The University of Hong Kong
- Anders Jacobsen Skanderup, Genome Institute of Singapore
- Bernett Lee, NTU
- Shuaicheng Li, The City University of Hong Kong
- Yu Li, The Chinese University of Hong Kong
- Fritz Sedlazeck, Baylor College of Medicine
- Hyun Uk Kim, Korea Advanced Institute of Science and Technology
- Chuanle Xiao, Sun Yat-sen University
- Can Yang, The Hong Kong University of Science and Technology
- Yuedong Yang, Sun Yat-sen University
- Kai Ye, Xi’an Jiao Tong University
- Xiangxiang Zeng, Hunan University

## PROGRAMME COMMITTEE MEMBERS

- Michael Gromiha, Bharathidasan University
- Kana Shimizu, Waseda University
- La Mai Chan, A* Star
- Souman Dey Kakon, Sher-e-Bangla Agricultural University
- Katarina Pinjusic, Harvard University
- Yuedong Yang, Sun Yat-sen University
- Marie Loh, Nanyang Technological University
- Jatin Joshi, University Of Delhi
- Xuefeng Cui, Shandong University
- Ruibang Luo, The University of Hong Kong
- Yu Li, Chinese University of Hong Kong
- Martin Frith, University of Tokyo
- Bernett Lee, Nanyang Technological University
- Nguyen Quoc Khanh Le, Taipei Medical University
- Lirong Cao, The Chinese University of Hong Kong
- Ting-Fung Chan, The Chinese University of Hong Kong
- Jian-Dong Huang, The University of Hong Kong
- Tsung Fei Khang, University of Malaya
- Jaebum Kim, Seoul National University
- Sarah Langley, Nanyang Technological University
- James Miller, Nanyang Technological University
- Kenta Nakai, The University of Tokyo
- Kumar Selvarajoo, A*STAR
- Lu Zhang, Hong Kong Baptist University
- Jatin Joshi, University Of Delhi
- Tatsuya Akutsu, Kyoto University
- Nathan Harmston, Cardiff University
- Alvin Ng, Nanyang Technological University
- Ka-Chun Wong, City University of Hong Kong
- Yoshihiro Yamanishi, Nagoya University
- Jesper Jansson, Kyoto University
- Hiu Fung Yip, Hong Kong Baptist University
- Souman Dey Kakon, Sher-e-Bangla Agricultural University
- Govinda Rao Dabburu, University of Delhi
- Limsoon Wong, National University of Singapore

